# Correction: Comparative Immunogenicity of HIV-1 gp160, gp140 and gp120 Expressed by Live Attenuated Newcastle Disease Virus Vector

**DOI:** 10.1371/annotation/670112f8-b4fd-4622-8a8c-203dc647f807

**Published:** 2014-01-29

**Authors:** Sunil K. Khattar, Sweety Samal, Celia C. LaBranche, David C. Montefiori, Peter L. Collins, Siba K. Samal

An error occurred in Figure 9; Figure 9B is identical to Figure 9A. Please see the corrected version of Figure 9B here: http://plosone.org/corrections/pone.0078521.g009b.cn.tif

